# Artery Tertiary Lymphoid Organs Control Multilayered Territorialized Atherosclerosis B-Cell Responses in Aged *ApoE*^*−/−*^ Mice

**DOI:** 10.1161/ATVBAHA.115.306983

**Published:** 2016-05-25

**Authors:** Prasad Srikakulapu, Desheng Hu, Changjun Yin, Sarajo K. Mohanta, Sai Vineela Bontha, Li Peng, Michael Beer, Christian Weber, Coleen A. McNamara, Gianluca Grassia, Pasquale Maffia, Rudolf A. Manz, Andreas J.R. Habenicht

**Affiliations:** From the Cardiovascular Research Center, Department of Medicine (P.S., C.A.M.), Department of Surgery (S.V.B.), University of Virginia, Charlottesville; Institute for Immunology (D.H.) and Institute for Cardiovascular Prevention (C.Y., S.K.M., C.W., A.J.R.H.), Ludwig-Maximilians-University, Munich, Germany; Institute of Molecular Immunology, Helmholtz-Zentrum München, Munich, Germany (D.H.); Department for Information Technology, University of Jena, Jena University Hospital, Jena, Germany (M.B.); Centre for Immunobiology, Institute of Infection, Immunity and Inflammation, College of Medical, Veterinary and Life Sciences, University of Glasgow, Glasgow, United Kingdom (G.G., P.M.); BHF Centre for Excellence in Vascular Science and Medicine, College of Medical, Veterinary and Life Sciences, University of Glasgow, Glasgow, United Kingdom (P.M.); Department of Pharmacy, University of Naples Federico II, Naples, Italy (P.M.); Institute for Systemic Inflammation Research, University of Lübeck, Lübeck, Germany (R.A.M.); and Department of Traditional Chinese Medicine, Medical College of Xiamen University, Xiamen University, Xiamen, China (L.P.).

**Keywords:** aging, atherosclerosis, B-lymphocytes, germinal center, inflammation

## Abstract

**Objective—:**

Explore aorta B-cell immunity in aged apolipoprotein E-deficient (*ApoE*^*−/−*^) mice.

**Approach and Results—:**

Transcript maps, fluorescence-activated cell sorting, immunofluorescence analyses, cell transfers, and Ig-ELISPOT (enzyme-linked immunospot) assays showed multilayered atherosclerosis B-cell responses in artery tertiary lymphoid organs (ATLOs). Aging-associated aorta B-cell–related transcriptomes were identified, and transcript atlases revealed highly territorialized B-cell responses in ATLOs versus atherosclerotic lesions: ATLOs showed upregulation of bona fide B-cell genes, including Cd19, Ms4a1 (Cd20), Cd79a/b, and Ighm although intima plaques preferentially expressed molecules involved in non–B effector responses toward B-cell–derived mediators, that is, Fcgr3 (Cd16), Fcer1g (Cd23), and the C1q family. ATLOs promoted B-cell recruitment. ATLO B-2 B cells included naive, transitional, follicular, germinal center, switched IgG1^+^, IgA^+^, and IgE^+^ memory cells, plasmablasts, and long-lived plasma cells. ATLOs recruited large numbers of B-1 cells whose subtypes were skewed toward interleukin-10^+^ B-1b cells versus interleukin-10^−^ B-1a cells. ATLO B-1 cells and plasma cells constitutively produced IgM and IgG and a fraction of plasma cells expressed interleukin-10. Moreover, *ApoE*^*−/−*^ mice showed increased germinal center B cells in renal lymph nodes, IgM-producing plasma cells in the bone marrow, and higher IgM and anti–MDA-LDL (malondialdehyde-modified low-density lipoprotein) IgG serum titers.

**Conclusions—:**

ATLOs orchestrate dichotomic, territorialized, and multilayered B-cell responses in the diseased aorta; germinal center reactions indicate generation of autoimmune B cells within the diseased arterial wall during aging.

## Abstract 2

Supplemental Digital Content is available in the text.

Beyond their ability to produce antibodies,^1^ B cells produce proinflammatory or anti-inflammatory cytokines,^2,3^ present antigen to T cells,^4^ and regulate B- and T-cell responses.^5^ Mature naive bone marrow (BM)–derived B-2 cells home into secondary lymphoid organs (SLOs) where they undergo somatic hypermutation and affinity maturation in germinal centers (GCs). Antigen-experienced B-2 cells either become short-lived plasma cells (PCs) residing in SLOs or they develop into long-lived PCs that largely home to the BM.^6–8^ By contrast, the majority of B-1 cells are located in the peritoneal cavity (PerC) and pleural cavities where they form a pool of quiescent innate B cells. On migration to inflammatory tissues, B-1 cells become activated and self-renew to carry out T-cell–independent protective immune responses.^9–12^ Recent reports showed differential effects of B-cell subsets in atherosclerosis^13–24^ with antiatherogenic effects of B-1 cells and proatherogenic effects of B-2 cells.^25–27^ In addition to SLOs and the BM, B-cell responses may be organized in artery tertiary lymphoid organs (ATLOs) in apolipoprotein E-deficient (*ApoE*^*−/−*^) mice.^28,29^ Here, we report on local aorta as opposed to systemic B-cell responses during aging.

## Materials and Methods

Materials and Methods are available in the online-only Data Supplement.

## Results

### Aorta B-Cell Transcripts During Aging

**Figure 1.**
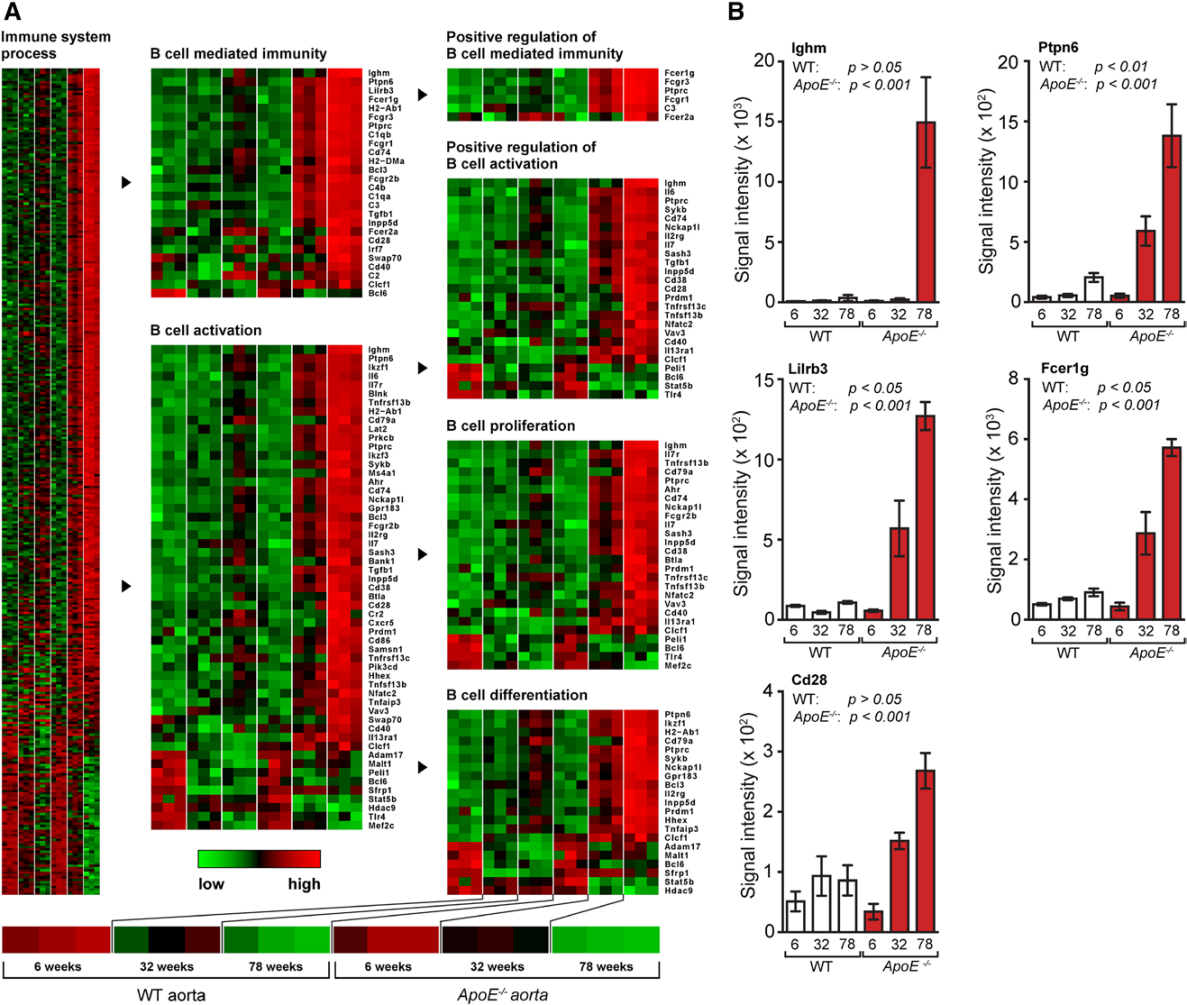
Aging-associated changes in aorta B-cell immunity. **A**, Age-associated transcript profiles of wild-type (WT) and *ApoE*^*−/−*^ aorta of 6-, 32-, and 78-week-old mice (3 mice per genotype per age group). Transcripts in gene ontology terms immune system process, B-cell–mediated immunity, B-cell activation, positive regulation of B-cell–mediated immunity, positive regulation of B-cell activation, B-cell proliferation, and B-cell differentiation are displayed as heatmaps. **B**, Expression of selected genes in aorta from WT and *ApoE*^*−/−*^ mice at 6, 32, and 78 weeks; n=3 mice per genotype per age group. Results represent mean±SEM. Analyses were performed using ANOVA with Benjamini–Hochberg correction. Absolute numbers of signal intensities and statistics are reported in Table I in the online-only Data Supplement.

MIAME (minimum information about a microarray experiment)-compliant microarrays were prepared as described^30,31^; data were deposited in the National Center for Biotechnology Information (http://www.ncbi.nlm.nih.gov/) and the gene ontology (http://www.geneontology.org/) data banks (accession GSE40156).^30,32^ To determine if B-cell–related gene expression changes with aging, microarrays of aortas, SLOs, and blood from wild-type (WT) and *ApoE*^*−/−*^ mice were compared. B-cell–related genes were altered in WT aortas during aging (Table I in the online-only Data Supplement). However, there were much more pronounced changes in *ApoE*^*−/−*^ when compared with WT aortas. Expression kinetics of some of these genes correlated with the kinetics of ATLO formation^32,33^ (Figure 1; Table I in the online-only Data Supplement). B-cell transcriptomes contained genes that were expressed exclusively by B cells and a majority of genes that respond to B-cell–derived molecules yielding a complex B-cell immunity–related gene map (Figure 1; Table I in the online-only Data Supplement). Examples of the magnitude of B-cell immunity–related transcripts in *ApoE*^*−/−*^ aortas include a 135-fold increase of Ighm (IgM constant region), a 29-fold increase in Ptpn6 (protein tyrosine phosphatase, nonreceptor type 6; SHP1) regulating the IgM repertoire, a 23-fold increase in the immunosuppressive Lilrb3 (leukocyte immunoglobulin-like receptor, subfamily B with transmembrane and immunoreceptor tyrosine-based inhibitory motif domains), Fcer1g (Fc receptor, IgE, high-affinity I, γ-polypeptide), and Cd28 (CD28 antigen) expression that promotes PC survival (Figure 1; Table I in the online-only Data Supplement). In contrast, spleen- and blood-transcript maps were considerably smaller, and the extent of differential expression between WT and *ApoE*^*−/−*^ mice was much less pronounced (Figure I in the online-only Data Supplement). The majority of B-cell–associated genes in the spleen and blood were downregulated during aging in both WT and *ApoE*^*−/−*^ mice: Ptprc (B220; Cd45; protein tyrosine phosphatase, receptor type, C) involved in cell fate decisions of the B-cell receptor; Aicda (activation-induced cytidine deaminase) regulating somatic hypermutation and Ig class switching; Sykb (spleen tyrosine kinase) participating in B-memory cell survival; Vav3 (Vav3 oncogene) mediating B-cell receptor responses; Tcf3 (transcription factor 3) controlling B-cell ontogeny; Foxp1 (forkhead box p1) impacting B-cell survival; and Malt1 (Malt1 paracaspase) participating in B-cell malignancies. In summary, the spleen and blood gene maps suggested that age-associated changes largely mirrored B-cell senescence rather than genotype/hyperlipidemia-dependent changes (Figure I and Table I in the online-only Data Supplement).

### Transcript Maps Delineate the Territoriality of B-Cell–Related Immune Responses in the Aged *ApoE*^*−/−*^ Aorta

**Figure 2.**
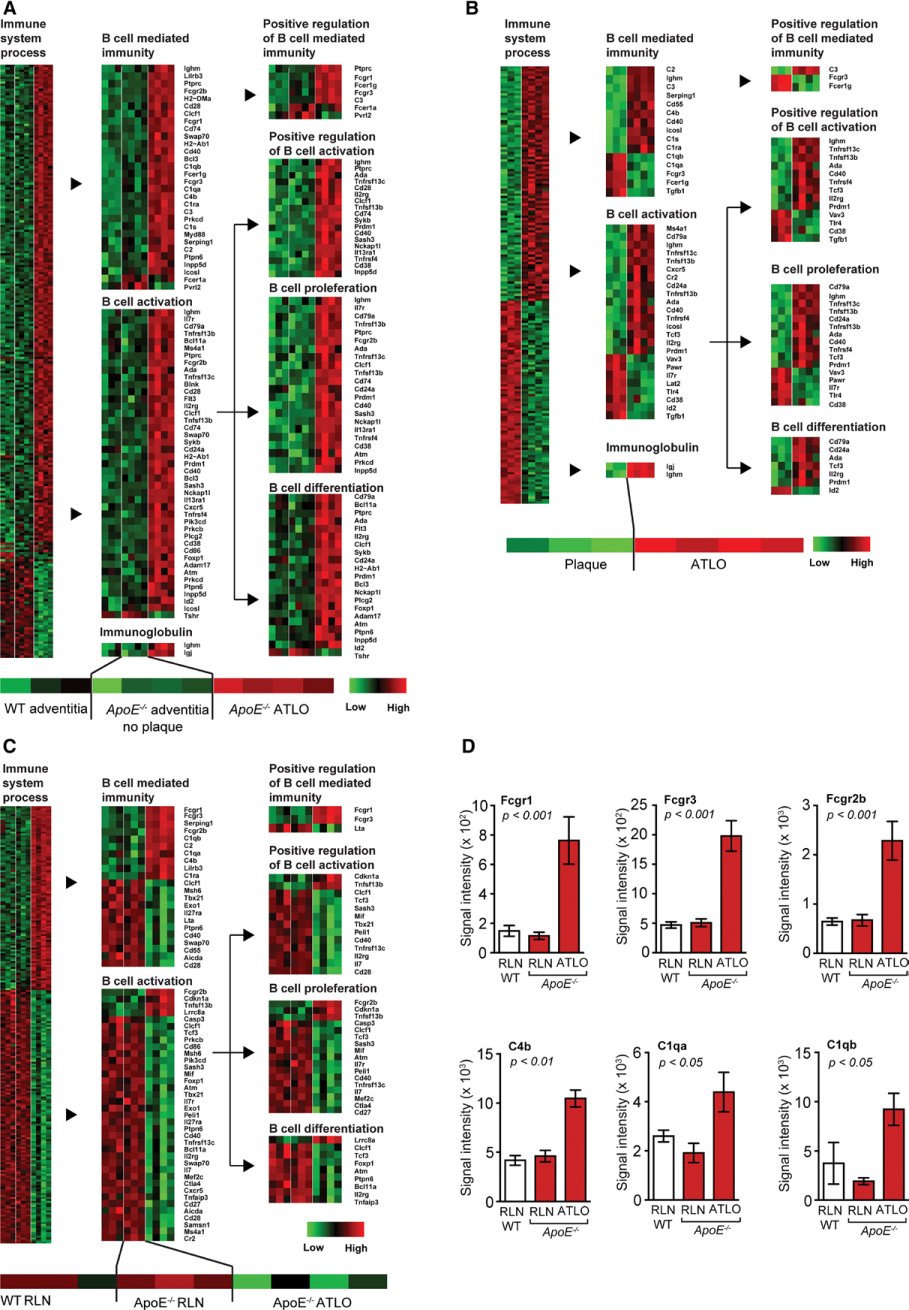
Aorta transcript maps reveal the specificity and territoriality of B-cell–related immune responses in artery tertiary lymphoid organs (ATLOs). **A**, Heatmaps of differentially expressed genes in the adventitia cluster (wild-type [WT], n=3; *ApoE*^*−/−*^, n=4). **B**, Plaque/ATLO cluster (for plaque, n=3; for ATLOs, n=4). **C**, Lymph node (LN) cluster (for WT and *ApoE*^*−/−*^ LNs, n=3; for ATLO, n=4); gene ontology terms immune system process, B-cell activation, B-cell–mediated immunity, immunoglobulin, positive regulation of B-cell–mediated immunity, positive regulation of B-cell activation, B-cell differentiation, and B-cell proliferation. **D**, Selected genes in the LN cluster. Results represent mean±SEM. Analyses were performed using ANOVA with Benjamini–Hochberg correction. Absolute numbers of signal intensities and statistics are reported in Table I in the online-only Data Supplement. RLN indicates renal LNs.

Laser capture microdissection aorta-derived tissues were obtained together with renal lymph nodes (RLNs) and spleen.^30,31^ B-cell–related genes were expressed at higher levels in ATLOs when compared with aorta adventitia segments from WT or *ApoE*^*−/−*^ mice without plaques (Figure 2A; Table I in the online-only Data Supplement). In the adventitia cluster, genes associated with B-cell survival, proliferation, differentiation, and activation, such as immunoglobulin genes (ighm), TACI (tnfrsf13b), B-cell activating factor receptor (tnfrsf13c), CD40 antigen (cd40), histocompatibility 2, class II antigen A, β-1 (h2-ab1), complement components (c1qb), and Myd88 (myd88) were robustly expressed in adventitial regions adjacent to plaques compared with adventitia in regions with no plaques (Figure 2A; Table I in the online-only Data Supplement). Moreover, the adventitia adjacent to plaques contained transcripts coding for Igj chain (immunoglobulin joining chain; Igj) involved in somatic hypermutation and memory B-cell development; CD79a (immunoglobulin-associated α; Ly54) involved in B-cell receptor signaling; and Ms4a1 (CD20) controlling T-cell–dependent humoral immunity (Figure IIA in the online-only Data Supplement). The plaque–ATLO cluster markedly expressed Cd19 (CD19 antigen) in ATLOs involved in B-cell maturation, Cd20, Igj chain, Igm, and Cd79a/b (Figure 2B; Figure IIB in the online-only Data Supplement). In addition, the plaque–ATLO B-cell cluster^30,31^ showed functional separation in B-cell–related genes in ATLOs versus plaques: bona fide B-cell genes displayed strong expression in ATLOs versus low expression in plaques. For example, Ighm, cd19, ms4a1 (cd20), Igj, and cd79a/b were expressed manifold higher in ATLOs when compared with plaques, which expressed genes that respond to B-cell products (Figure 2A; Figure IIB and Table I in the online-only Data Supplement). In contrast, the transcript atlas showed almost identical levels of B-cell–related genes in WT versus *ApoE*^*−/−*^ spleens, RLNs, and blood (Figure I in the online-only Data Supplement; Figure 2C and 2D). It is also noticeable that the LN–ATLO cluster shows a comparably higher expression in ATLOs versus LNs of innate immune response genes, such as fcgr1, fcgr2b, fcgr3, c4b, and the c1q family, indicating ongoing inflammation in ATLOs (Figure 2C and 2D).

### ATLO B-2 Subtypes Suggest Antigen-Specific GC Reactions

**Figure 3.**
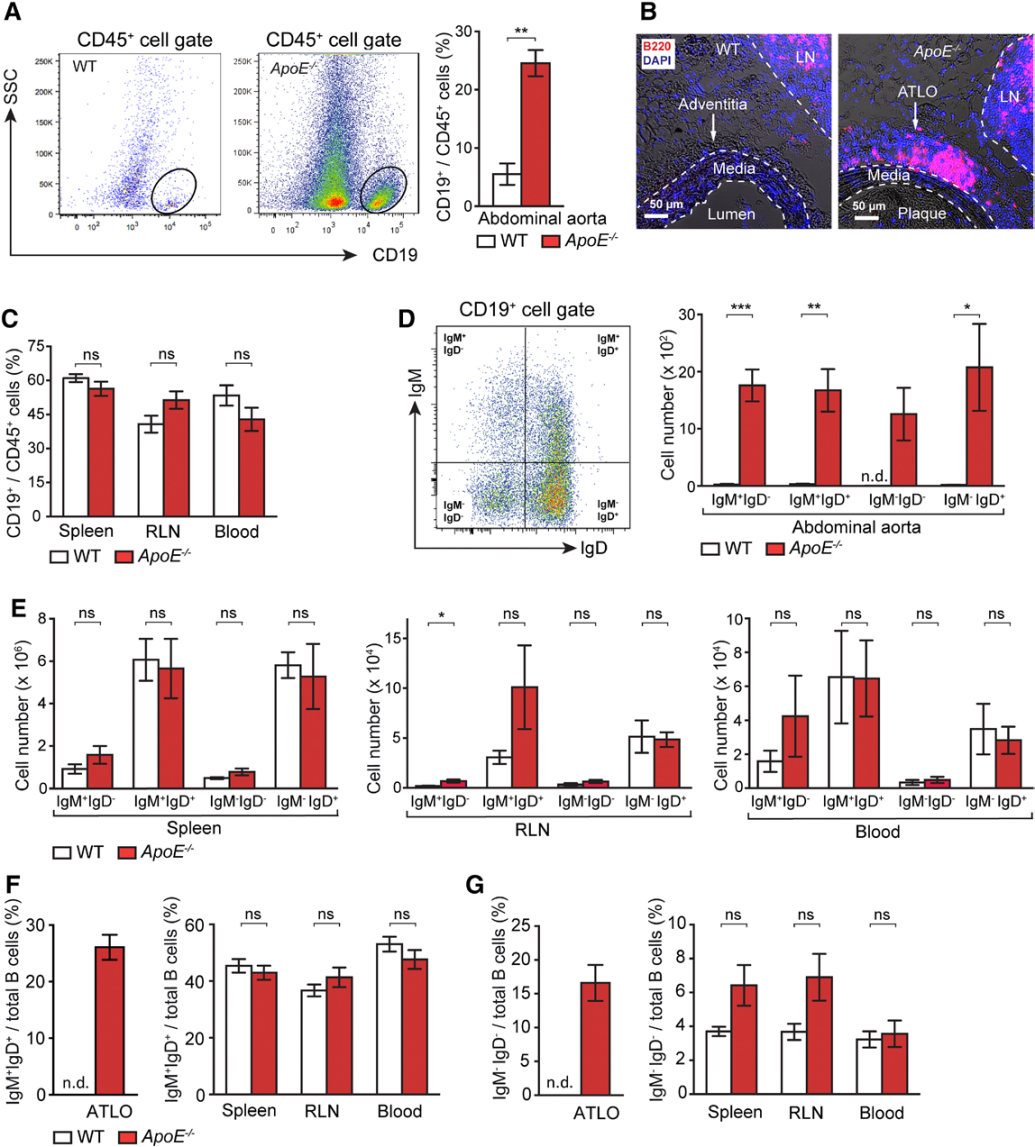
Artery tertiary lymphoid organs (ATLOs) harbor a diverse repertoire of B-cell subsets. **A**, Flow cytometry of CD19^+^ B cells of total CD45^+^ leukocytes in abdominal aorta of 80-week-old wild-type (WT) and *ApoE*^*−/−*^ mice (WT, n=11; *ApoE*^*−/−*^, n=10). **B**, Immunofluorescence staining with anti-B220 antisera shows B cells in ATLOs and lymph nodes (LNs) but none in the media (M) or plaque (P) or in WT adventitia. **C**, B cells in the spleen, renal LNs (RLNs), and blood of WT and *ApoE*^*−/−*^ mice (WT, n=8; *ApoE*^*−/−*^, n=6). Flow cytometric analysis of IgM^+^IgD^−^, IgM^+^IgD^+^, IgM^−^IgD^−^, and IgM^−^IgD^+^ B cells per total CD19^+^ B cells in abdominal aorta (**D**) and spleen, RLN, and blood of 80-week-old WT and *ApoE*^*−/−*^ mice (WT n=4; *ApoE*^*−/−*^ n=5; **E**). Percentages of IgM^+^IgD^+^ B cells (**F**) and IgM^−^IgD^−^ B cells per total B cells in ATLOs, spleen, RLN, and blood of age-matched WT and *ApoE*^*−/−*^ mice (**G**). Results represent mean±SEM; *P<0.05, **P<0.01, and ***P<0.001; 2-sided unpaired Student *t* test. n indicates the number of experiments; n.d., not detectable; ns, not significant; and SSC, side scatter.

B cells present in the aorta of aged *ApoE*^*−/−*^ mice predominantly reside in ATLOs, whereas they cannot be observed in plaques of young WT or *ApoE*^*−/−*^ mice.^30,32,33^ Fluorescence-activated cell sorting (FACS) analyses of B cells revealed the magnitude of differences in ATLOs and WT adventitia; and B220 immunostaining confirmed that B cells are located in ATLOs and in the adjacent draining LNs but none in WT adventitia or plaques (Figure 3A and 3B). Considerable numbers of T-/B-cell clusters referred to as fat-associated lymphoid clusters were observed in paraaortic adipose tissue of aged *ApoE*^*−/−*^ mice and numerous small paraaortic LNs containing B cells lined the tissue adjacent to the adventitia (not shown). There were no differences in the frequency of B cells in SLOs or blood of WT versus *ApoE*^*−/−*^ mice (Figure 3C). To obtain evidence for an ongoing GC reaction in ATLOs, CD19, IgM, and IgD antisera together with FACS gating for 4 different populations from CD19^+^ B cells were used (Figure 3D). IgM^+^/IgD^−^, IgM^+^/IgD^+^, IgM^−^/IgD^−^, and IgM^−^/IgD^+^ B cells were identified in abdominal but not thoracic aorta segments: IgM^+^/IgD^−^ cells represent either immature or transitional B cells (also referred to as T-1 cells) representing the earliest B-cell stage present outside the BM or these cells may represent B-1 B cells^34^; IgM^+^/IgD^+^ and IgM^−^/IgD^+^ cells represent mature B-cell stages.^35,36^ Among mature IgD^+^ cells, IgM^−^/IgD^+^ are mature follicular B-2 cells.^37^ IgM^−^/IgD^−^ cells represent either switched Ig^+^ B cells, GC B cells that have transiently lost Ig expression when undergoing hypermutation of their Ig genes or GC-derived memory B cells.^34,38^ None of the subsets were found in the abdominal aorta of WT mice (Figure 3D). WT and *ApoE*^*−/−*^ SLOs and blood revealed equivalent numbers of these subsets with the exception of an increase in transitional IgM^+^/IgD^−^ B cells in RLNs of *ApoE*^*−/−*^ versus WT mice (Figure 3E). We determined the percentages of IgM^+^/IgD^+^ or switched Ig^+^ B cells in SLOs, blood, WT aortas, and ATLOs. SLO and blood IgM^+^/IgD^+^ and switched Ig^+^ B cells were similar in WT and *ApoE*^*−/−*^ SLOs (Figure 3F and 3G). Although undetectable in WT adventitia, the percentage of IgM^+^/IgD^+^ B cells in ATLOs approached that in SLOs (Figure 3F). However, the percentage of switched Ig^+^ B cells in ATLOs exceeded those in SLOs or blood (Figure 3G). We determined the number of B-1 cells in the PerC and of plasmablasts and PCs in the abdominal aorta, spleen, and RLNs of *ApoE*^*−/−*^ mice (Figure III in the online-only Data Supplement). No change in B-1 B cell subtypes was observed in the PerC of WT versus *ApoE*^*−/−*^ mice (Figure IIIA in the online-only Data Supplement). Moreover, aged *ApoE*^*−/−*^ abdominal aortas, spleens, and RLNs contained plasmablasts and PCs; some of which expressed interleukin (IL)-10 (Figure IIIB in the online-only Data Supplement).

### ATLOs Harbor GC B Cells and IgG1^+^, IgA^+^, and IgE^+^ Memory Cells

**Figure 4.**
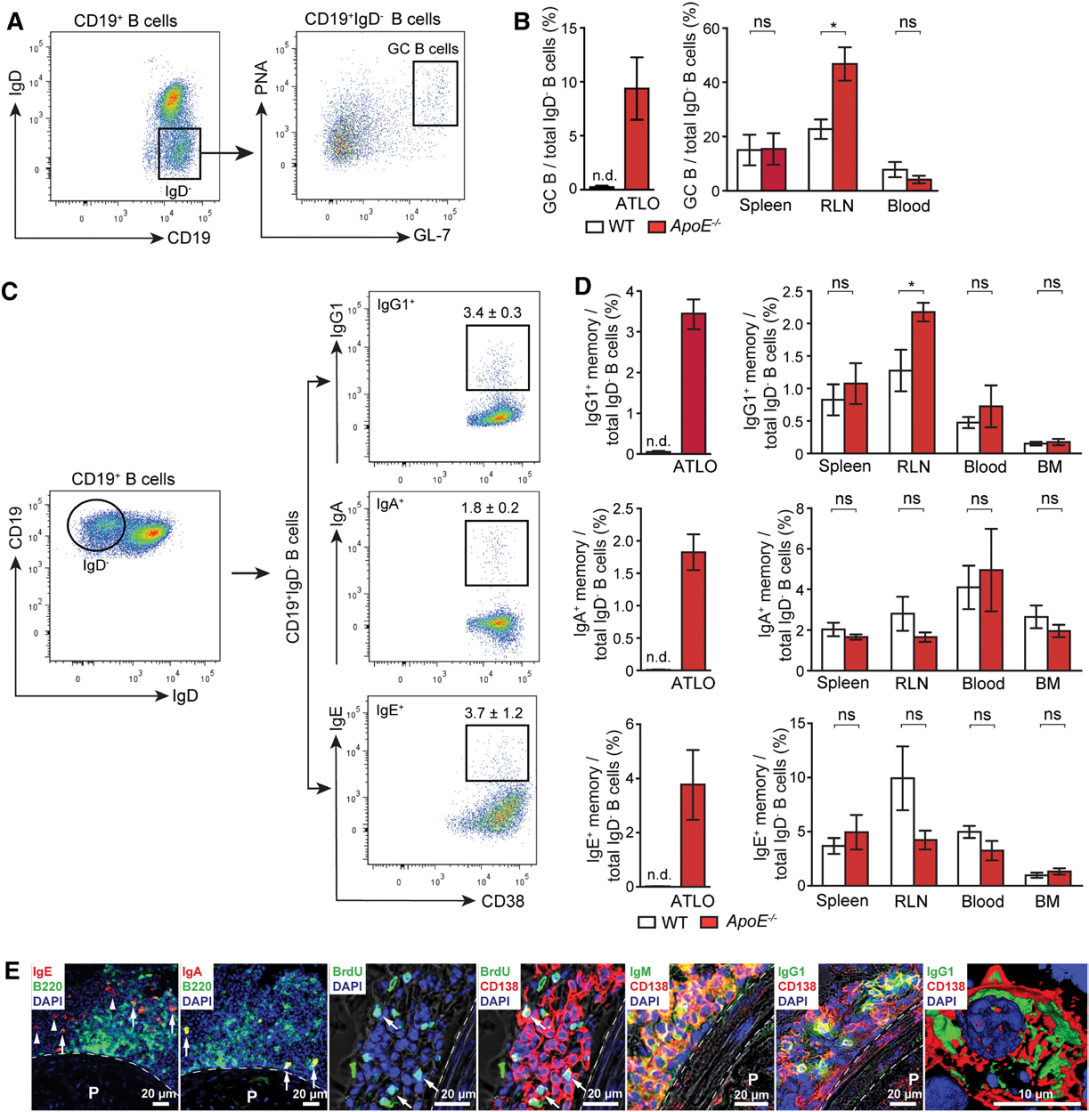
Artery tertiary lymphoid organs (ATLOs) contain B cells that participate in humoral immune responses. **A**, IgD^−^ B cells gated from CD19^+^ total B cells were evaluated for PNA^+^/GL-7^+^ (germinal center [GC] B cells) in ATLOs. **B**, GC B cells in ATLOs and SLOs were quantified (wild-type [WT], n=4; *ApoE*^*−/−*^, n=5). **C**, IgG1^+^ (IgG1^+^/CD38^+^), IgA^+^ (IgA^+^/CD38^+^), and IgE^+^ (IgE^+^/CD38^+^) memory B cells were gated from total CD19^+^/IgD^−^ B cells. **D**, Quantification of IgG1^+^, IgA^+^, and IgE^+^ memory B cells in ATLOs, SLOs, blood, and bone marrow (BM; WT, n=4; *ApoE*^*−/−*^, n=4). Results represent mean±SEM; *P<0.05, 2-sided unpaired Student *t* test. **E**, Immunofluorescence data of IgE^+^ memory B cells (IgE^+^/B220^+^ indicated with arrows and IgE^+^/B220^−^ cells indicated with arrow heads), IgA^+^ memory B cells (IgA^+^/B220^+^), long-lived plasma cells (PCs; CD138^+^/BrdU^−^) and short-lived PCs (CD138^+^/BrdU^+^; white arrow), IgM- (IgM^+^/CD138^+^), and IgG1- (IgG1^+^/CD138^+^) producing PCs in ATLOs. Dotted line outlines media. n indicates the number of experiments; n.d., not detectable; ns, not significant; P, plaque, and RLN, renal lymph node.

Naive B cells in SLOs enter GCs to undergo a GC reaction involving somatic hypermutation and affinity maturation of their B-cell receptors. ATLO GC B cells were identified by FACS (IgD^−^/PNA^+^/GL-7^+^): they were undetectable in WT aortas but ranged at ≈9% of all IgD^−^ B cells in ATLOs (Figure 4A and 4B). Their number was similar in WT and *ApoE*^*−/−*^ spleen and blood although they were more abundant in *ApoE*^*−/−*^ when compared with WT RLNs (Figure 4B). We sought evidence for isotype-switching using FACS analyses. Surprisingly, we observed significant numbers of CD19^+^/IgD^−^/IgG1^+^, CD19^+^/IgD^−^/IgA^+^, and CD19^+^/IgD^−^/IgE^+^ B cells in ATLOs (Figure 4C and 4D). Although class switching is not restricted to GCs, the presence of GCs and cells that class switched to T-dependent Ig subclasses, such as IgG1, suggests that these cells resemble memory B cells. Intriguingly, the percentage of IgD^−^ B cells that class switched to IgG1 was significantly greater than those in the spleen, RLNs, BM, or blood (Figure 4D). In contrast, there were equivalent percentages of IgG1^+^ B cells in the spleen, BM, and blood of WT versus *ApoE*^*−/−*^ mice. Consistent with rare ATLO formation in the thoracic aorta,^32^ no GC B cells or class switched B cells were observed there (not shown). These data provide evidence for a disease-specific antigen-dependent B-2 B cell maturation pathway in ATLOs.

### Short-Lived and Long-Lived PCs in ATLOs

Long-lived PCs are major constituents of humoral memory. Long-lived PCs preferentially home to the BM, whereas short-lived PCs remain within SLOs. Nothing is known about PCs in atherosclerosis. As long-lived PCs survive for long periods of time in the BM,^39^ we determined the composition of ATLO PC subtypes.^8^ Both long-lived and short-lived PCs were observed in ATLOs (Figure 4E).^40,41^ Moreover, survival factors for long-lived PCs, including CXCL12, B-cell activating factor,^39^ and others, are markedly expressed in ATLOs^30,32^ (Table I in the online-only Data Supplement).

### ATLOs Promote B-2 and B-1 Cell Recruitment Into the Arterial Wall

**Figure 5.**
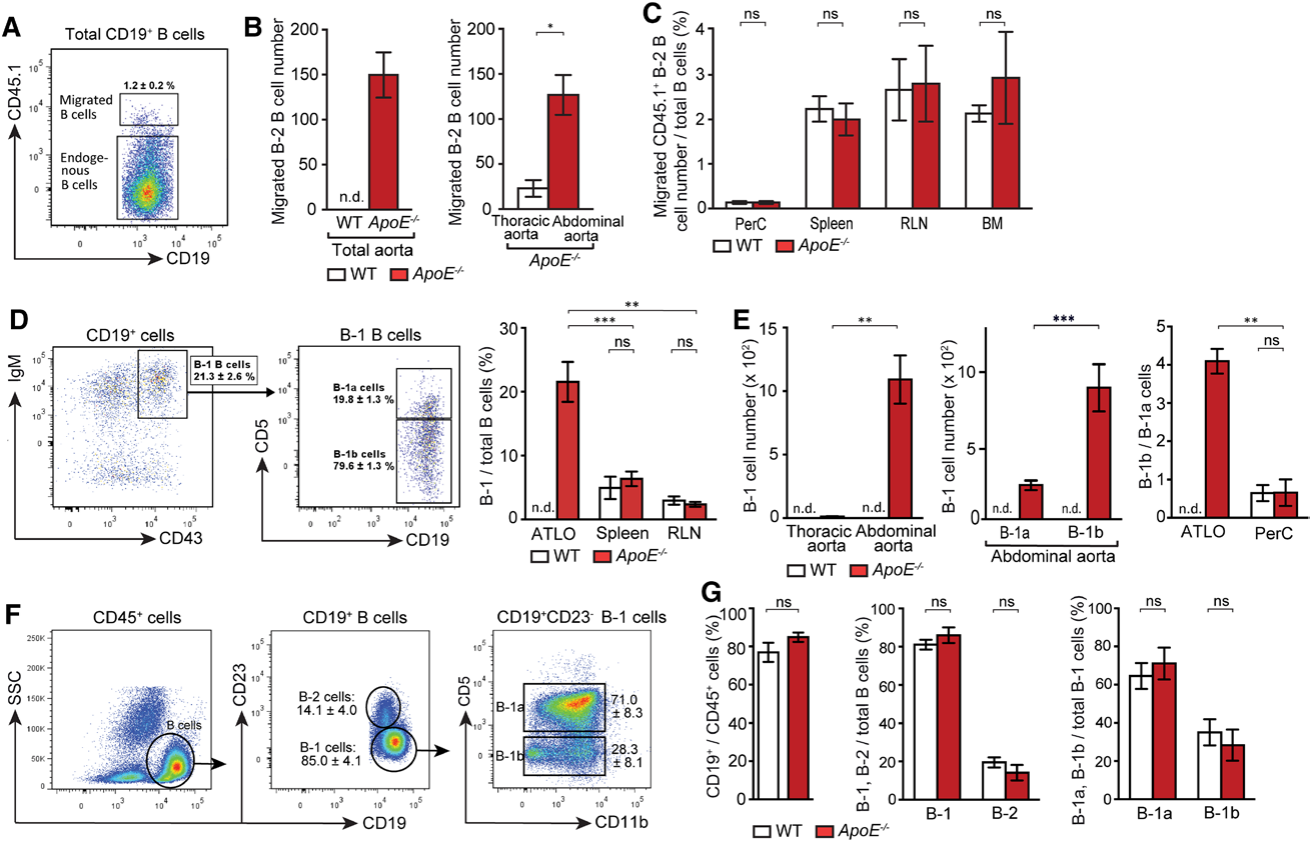
Artery tertiary lymphoid organs (ATLOs) promote B-2 B-cell recruitment into the abdominal aorta, skewing of ATLO B-1 cells toward B-1b cells. Fluorescence-activated cell sorting (FACS)–purified Ly5.1^+^ CD19^+^/CD43^−^ B-2 cells (purity, >98%) were adoptively transferred via tail vein injection into aged wild-type (WT) or *ApoE*^*−/−*^ mice. Thirty-six hours later, Ly5.2^+^ recipient mice were analyzed for B-2 cell migration into ATLOs or thoracic aorta segments. **A**, Migrated B-2 B cells were gated from total B cells in ATLOs. **B**, Quantification of migrated Ly5.1^+^ B-2 B cells in aorta. **C**, Peritoneal cavity (PerC), spleen, renal lymph nodes (RLNs), and bone marrow (BM). Results represent mean±SEM; *P<0.05, 2-sided unpaired Student *t* test. WT, n=3; *ApoE*^*−/−*^, n=3. B-1 cells selectively accumulate in ATLOs. **D**, IgM^hi^/CD43^+^ B-1 cells were gated from CD19^+^ B cells, and CD5^+^ B-1a and CD5^−^ B-1b cells were gated from total B-1 cells in ATLOs and the percentage of B-1 cells from total B cells were quantified in ATLOs and SLOs. **E**, Absolute numbers of B-1 cells were quantified in aortic segments in WT and *ApoE*^*−/−*^ mice. The ratio of B-1b/B-1a B cells in ATLOs compared with that in PerC of WT and *ApoE*^*−/−*^ mice. FACS plots show the gating strategy for B-cell subpopulations in PerC (**F**) and their frequencies of B cells in CD45^+^ cells, B-1, and B-2 cells in total B cells, B-1a, and B-1b cells in B-1 cells were compared between WT and *ApoE*^*−/−*^ mice (**G**). Results represent mean±SEM; **P<0.01 and ***P<0.001; 2-sided unpaired Student *t* test with Bonferroni–Holm correction. WT and *ApoE*^*−/−*^, n=5–6. n indicates the number of experiments; n.d., not detectable; ns, not significant; and SSC, side scatter.

To determine B-cell recruitment by ATLOs, we adoptively transferred Ly5.1 B-2 cells to aged Ly5.2 WT or *ApoE*^*−/−*^ mice. After 36 hours, B-2 cells had migrated predominantly to the abdominal aorta of *ApoE*^*−/−*^ mice (Figure 5A and 5B) although none were recruited to WT aortas. Comparably low but similar numbers of B-2 cells were recruited into the PerCs of WT and *ApoE*^*−/−*^ mice. There was no difference in B-cell recruitment into the spleen, RLNs, and BM of WT versus *ApoE*^*−/−*^ mice (Figure 5C). Similar data were obtained with B-1 cells (not shown).

### B-1 Cells Accumulate in ATLOs and Are Skewed Toward B-1b Cells

B-1 cells are predominantly located in body cavities.^42,43^ Recent studies showed that B-1a cells reside in the aorta perivascular tissue of young *ApoE*^*−/−*^ mice.^22^ To determine if B-1 cells are located in the aged aorta adventitia, we performed FACS analyses. A high percentage of all B cells, that is, ≈21%, in ATLOs were B-1 cells (Figure 5D), and their relative contribution to all B cells exceeded that in the spleen and RLNs by a large margin (Figure 5D). The reason for B-1 B-cell accumulation is most likely the high expression of CXCL13 in ATLOS. Numbers of total B-1 cells in ATLOs are comparable with that of IgM^+^/IgD^−^ cells, indicating that most IgM^+^/IgD^−^ cells found in this compartment are B-1 cells. The abdominal aorta harbored considerably higher numbers of B-1 cells when compared with the thoracic aorta (Figure 5E). The B-1 subtype composition was aberrant as we observed a high number of B-1b versus B-1a cells, which dramatically differs from that relation in the PerC (Figure 5E).^9^ There was no significant difference in total B cells, B-2, B-1a, and B-1b cells in the PerC of aged WT and *ApoE*^*−/−*^ mice (Figure 5F and 5G).

### Majority of ATLO B-1b but Not B-2 Cells Express IL-10, PD-L1, FasL, and Transforming Growth Factor-β

**Figure 6.**
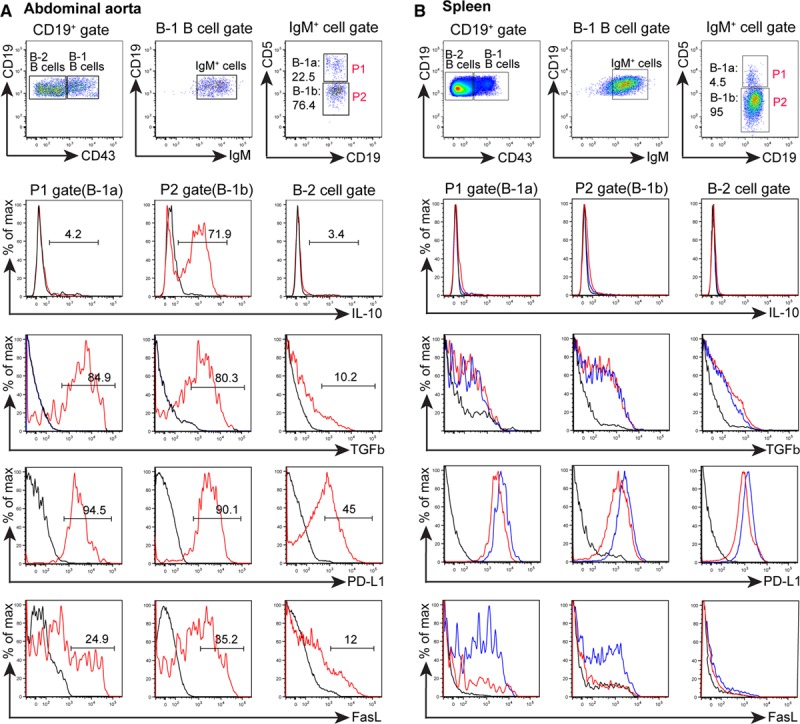
Artery tertiary lymphoid organ (ATLO) B-1 B cells show a predominant immunosuppressive IL-10^+^/PD-L1^+^/FasL^+^/TGFβ^+^ phenotype. Cell suspensions from individual aged *ApoE*^*−/−*^ mice. **A**, IL-10^+^, TGFβ^+^, PD-L1^+^, and FasL^+^ abdominal aorta B cells. **B**, *ApoE*^*−/−*^ (red) spleen (80- to 85-week old mice) and WT (blue); *ApoE*^*−/−*^ (n=3–4). B-1a, B-1b, and B-2 cell populations were gated and assayed for cytokine expression (or isotype control, black). Numbers designate frequencies of positive cells.

In view of skewing of ATLO B-1 cells toward the B-1b subtype (Figure 5D and 5E) and a recent report showing that B-1b cells protect against atherosclerosis,^21^ we searched for mechanisms of immunosuppression within the arterial wall. IL-10–producing B-1a rather than B-1b or B-2 cells were found in the PerC (Figure IIIA in the online-only Data Supplement). However, we observed that the majority (≈72%) of abdominal aorta B-1b cells produced IL-10 though a minor component of B-1a cells and a significant but low proportion of IL-10^+^ cells in the thoracic aorta (not shown). No or comparably low numbers of B-1a cells or B-2 cells expressed IL-10 (Figure 6A). Moreover, the frequency of IL-10^+^ B cells in ATLOs is higher than those of their counterparts in the spleen and RLNs of WT or *ApoE*^*−/−*^ mice (Figure 6B). Following a report that a subset of PCs secretes IL-10,^44^ we assessed IL-10 expression in PCs. A significant proportion of ATLO CD138^+^/CD19^+^ plasmablasts were IL-10^+^ PCs (Figure IIIB in the online-only Data Supplement). Similar PCs have been shown to suppress immune responses in disease models.^45^ We further assessed the phenotype of B-1 cells in the abdominal aorta. ATLO B-1 but to a much lesser extent B-2 cells expressed PD-L1, FasL, and transforming growth factor-β, indicating that these cells exert immunosuppressive functions (Figure 6A).

### Ig-Secreting Cells Accumulate in ATLOs

**Figure 7.**
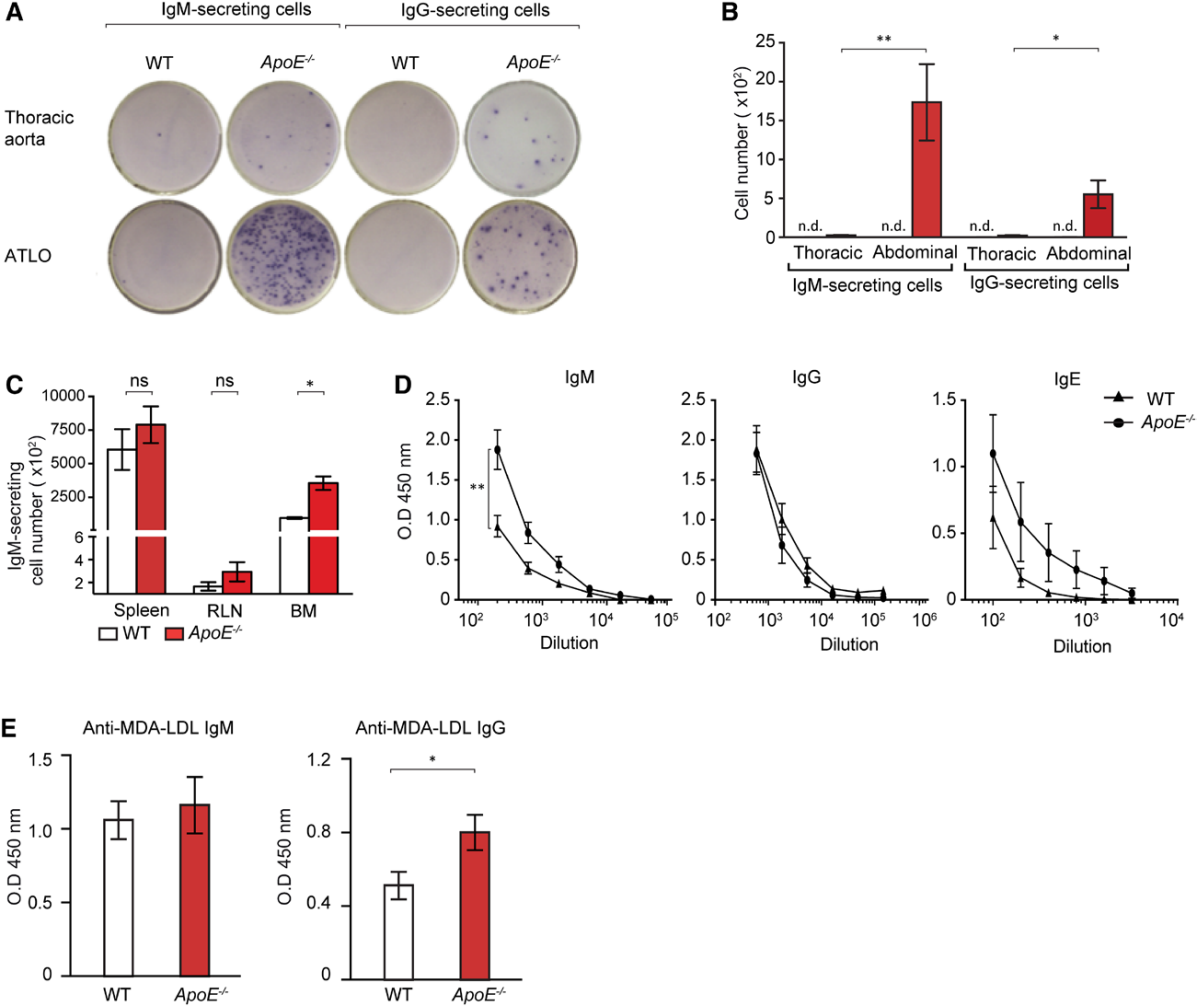
IgM- and IgG-secreting B cells are selectively located in artery tertiary lymphoid organs (ATLOs). **A**, ELISPOT (enzyme-linked immunospot) analyses of IgM- and IgG-secreting B cells in ATLOs and thoracic aorta segments. **B**, Quantification of IgM- and IgG-secreting cells in ATLOs versus thoracic aorta. **C**, Quantification of IgM-secreting cells in the spleen, renal lymph node (RLN) and bone marrow (BM) of age-matched wild-type (WT) and *ApoE*^*−/−*^ mice. **D**, Serum titers of IgM, IgG, and IgE in aged WT and *ApoE*^*−/−*^ mice. **E**, Anti–MDA-LDL IgM and anti–malondialdehyde-modified low-density lipoprotein (MDA-LDL) IgG serum titers (dilution factor 10 and 25, respectively) in aged WT and *ApoE*^*−/−*^ mice. Results represent mean±SEM; 2-sided unpaired Student *t* test; n=10 per genotype; *P<0.05 and **P<0.01. ns indicates not significant.

ELISPOT (enzyme-linked immunospot) experiments were performed. There were no constitutively IgM- and IgG-secreting cells in either the thoracic or abdominal aorta of WT mice (Figure 7A and 7B). Few IgM- and IgG-secreting cells were observed in the thoracic aorta of *ApoE*^*−/−*^ mice (Figure 7A and 7B). However, ATLOs contained abundant IgM- and IgG-secreting cells amounting to ≤80-fold increase of IgM-secreting B cells and a 24-fold increase in IgG-secreting B cells in the abdominal aorta (Figure 7A and 7B). Blood contains few (<10 cells per 0.5 mL of blood) IgM- or IgG-secreting cells (data not shown). In the spleen and RLNs, there was no difference in Ig-secreting cells between WT and *ApoE*^*−/−*^ mice (Figure 7C). However, IgM-secreting cells were higher in *ApoE*^*−/−*^ BM when compared with WT BM raising the possibility of a systemic PC response in *ApoE*^*−/−*^ mice. To examine a systemic B-cell response, we determined serum titers of IgM, IgG, and IgE, as well as anti–malondialdehyde-modified low-density lipoprotein (MDA-LDL) IgM and IgG. Aged *ApoE*^*−/−*^ mice had significantly higher levels of total IgM but not IgG or IgE levels when compared with aged WT mice (Figure 7D). Although anti–MDA-LDL IgM levels were not different, anti–MDA-LDL IgG levels were significantly higher in *ApoE*^*−/−*^ versus WT mice (Figure 7E).

## Discussion

These data identify ATLOs as the principal lymphoid tissue that orchestrates atherosclerosis B-cell immunity during aging of *ApoE*^*−/−*^ mice. Atherosclerosis ATLO B-cell responses are specific, robust, highly territorialized, multilayered, and include a comprehensive adaptive B-2 and a substantial aberrant innate B-1 cell component: ATLOs but not WT adventitia harbor an unusual set of class-switched IgG1^+^, IgA^+^, and IgE^+^ B cells, a significant number of IL-10^+^/PD-L1^+^/FasL^+^/TGFβ^+^ B-1b cells, and both short-lived and long-lived PCs, including a fraction of IL-10^+^ PCs. This body of data—together with our previous observation that B cells are major constituents of ATLO antigen-presenting cells^30^—reveal a yet unrecognized scenario of aorta atherosclerosis-specific B-cell immunity, which includes B effector cells, PCs, and several immunosuppressive B-cell subtypes (Figure IV in the online-only Data Supplement).

ATLO B-2 B-cell subtypes include transitional, follicular, GC, and IgG1^+^, IgA^+^, and IgE^+^ B cells—the latter representing class-switched B cells and PCs. These data are the first to suggest that (auto)antigen-dependent hypermutation, proliferation, affinity maturation, Ig class switching, memory cell generation, and differentiation into long-lived PCs may be carried out in the arterial wall. It is becoming evident that ATLOs provide a new paradigm of atherosclerosis-specific B-cell immunity and possibly autoimmunity: ATLO B-cell responses occur in aged animals, whereas aortas of young *ApoE*^*−/−*^ or young and aged WT mice do not show a significant aorta B-cell compartment.^30,46–48^ It should be pointed out, however, that this study falls short of proving antigen-specific ATLO-dependent autoimmune B-2 B-cell generation. In this regard, the observation of a considerable number of PCs in ATLOs deserves special attention: PCs may arise from B-1 cells, from B-2 cells via T-cell–independent mechanisms, or from B-2 cells via T-cell–dependent mechanisms.^49^ Further studies on the origin of aorta PCs seem warranted as the role of PCs in atherosclerosis remains unknown.

Our data demonstrate that local B-cell immune subsets can be distinguished from those in SLOs, the PerC, and the BM: their aberrant nature manifests itself by the presence of large numbers of IL-10^+^ B-1b cells, of short-lived and long-lived PCs, and of IL-10^+^ PCs. Possibly, our aged mice will allow to isolate B cells from ATLOs and SLOs to compare their B-cell receptor repertoire. Moreover, the accumulation of IgA^+^ and IgE^+^ B cells in the diseased aorta indicates links of atherosclerosis B-cell immunity to innate inflammatory leukocytes in plaques. IgA, IgE, and IgG act through either activating or inhibitory Fc receptors on virtually all innate immune cells, including macrophages.^50^ The expression of divergent Fc receptors raises the possibility that Fc receptors may be involved in the dichotomic control of inflammation within diseased arteries: Fcer1g (Cd23) is a high-affinity IgE receptor that is upregulated during aging, and Fcgr1 (Cd64), Fcgr2b (Cd32), and Fcgr3 (Cd16) are prominently expressed in ATLOs.

ATLOs contain multiple B-cell subtypes, including IgM^+^/IgD^−^, IgM^+^/IgD^+^, IgM^-^/IgD^−^, and IgM^−^/IgD^+^ B cells. ATLO IgM^+^/IgD^−^ and IgM^+^/CD43^+^ B cells may be B-1 cells. In addition, the presence of class-switched memory B cells suggests that some ATLO IgM^+^/IgD^−^ B cells may represent IgM^+^ memory B cells that have not undergone class switching. IgM^+^ memory B cells considerably contribute to the total population of all memory B cells.^51^ Whether the population of IgM^+^/IgD^−^ B cells within ATLOs also includes a fraction of immature or transitional B cells that represent the earliest B-cell stages that are found outside the BM is a possibility that deserves attention. Under physiological conditions, immature B cells immigrate from the BM and specifically home to splenic follicles to undergo differentiation into a transitional B-cell stage and finally either become mature B-2 or marginal zone B cells.^52^ This final B-cell maturation is accompanied by a shift of the B-cell receptor repertoire that includes counterselection against autoreactive cells that occurs in discrete and tightly controlled steps.^53^ Hence, it is tempting to speculate that immature B cells home to ATLOs to undergo differentiation into mature B cells in the absence of the proper control mechanisms acting in the spleen: this could allow for the generation of autoreactive atherosclerosis-specific B cells.

## Acknowledgments

We thank Dr Gompf (Leibniz Institute for Aging Research, Jena) for fluorescence-activated cell sorting.

## Sources of Funding

This work was funded by the German Research Council: HA 1083/15-4 to A.J.R. Habenicht; YI 133/2-1 to C. Yin; and MO 3054/1-1 to S.K. Mohanta; the German Centre for Cardiovascular Research (MHA VD1.2), SFB 1123/A1 and Z3, the European Research Council (AdG 249929) to C. Weber, by The British Heart Foundation: PG/12/81/29897 to P. Maffia and RE/13/5/30177; and the European Commission Marie Skłodowska-Curie Individual Fellowship 661369 to G. Grassia.

## Disclosures

None.

## Supplementary Material

## Highlights

- Artery tertiary lymphoid organs (ATLOs) orchestrate B-cell responses in the diseased aorta.
- ATLOs promote B-1 and B-2 cell recruitment into the arterial wall.
- ATLOs contain germinal center B cells and both short-lived and long-lived plasma cells.
- ATLOs harbor an aberrant set of B-1 cells whose subtype is skewed toward B-1b cells.
- A fraction of ATLO B cells produce IgM or IgG.
