# Supplementary material for: Artery Tertiary Lymphoid Organs Control Multilayered Territorialized Atherosclerosis B-Cell Responses in Aged ApoE−/− Mice
Source: Arterioscler Thromb Vasc Biol. 2016 May 25;36(6):1174–85. doi: 10.1161/ATVBAHA.115.306983 (PMC4894775; doi:10.1161/ATVBAHA.115.306983)
Supplement: Supplementary file 1 [file atv-36-1174-s001.pdf]

Supplement Material

Artery Tertiary Lymphoid Organs Control Multi-layered Territorialized Atherosclerosis B Cell Responses in Aged *ApoE*$^{-/-}$ Mice

Prasad Srikakulapu *et al*.

Supplemental Figures

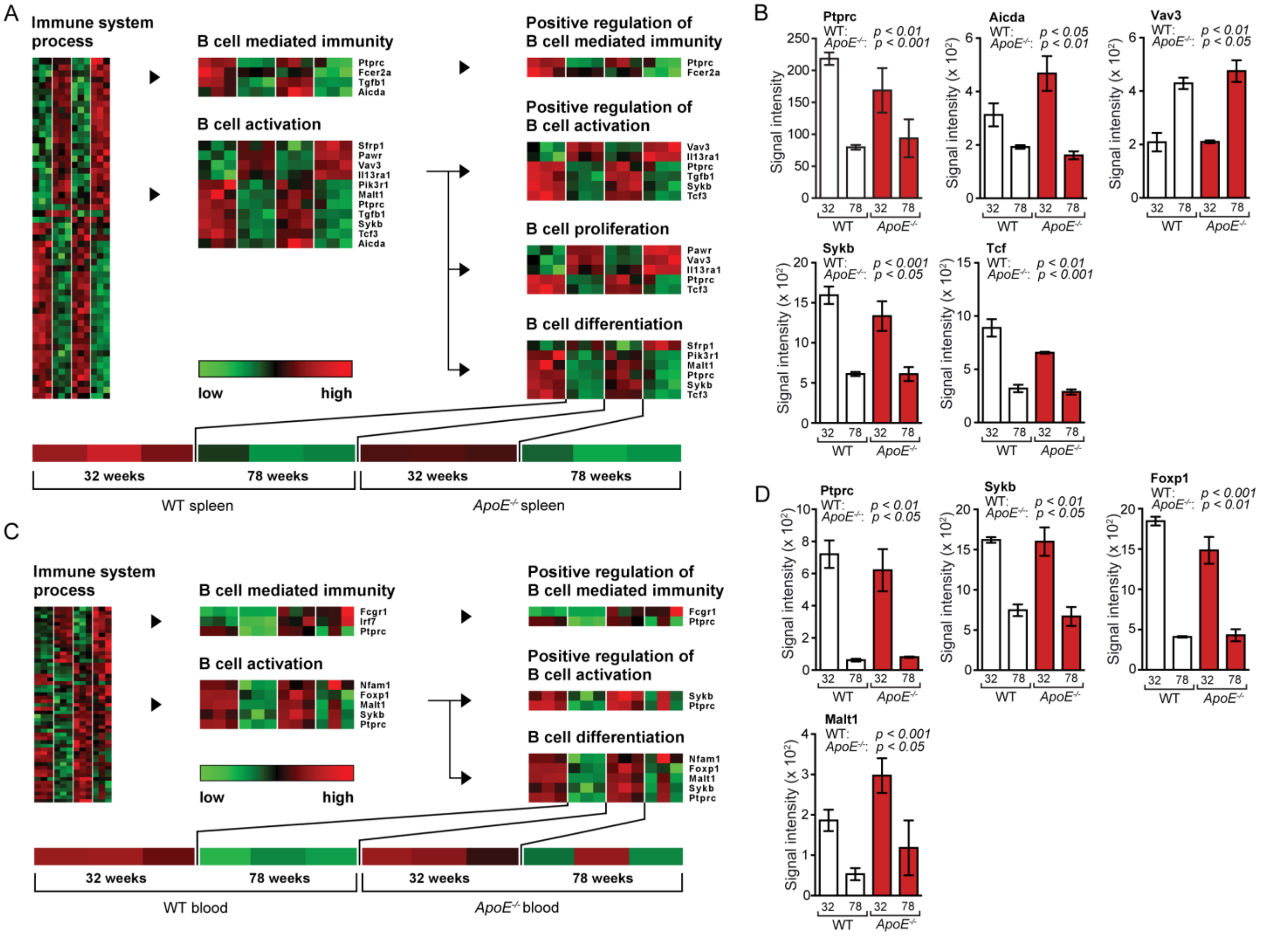

Figure SI: Age-associated B cell gene expression in spleen and blood of WT and *ApoE*$^{-/-}$ mice. Age-associated transcript profiles were obtained for total spleen and blood from 32 and 78 weeks old WT and *ApoE*$^{-/-}$ mice (n=3 mice per genotype per age group). Transcripts in GO terms Immune system process, B cell mediated immunity, B cell activation, positive regulation of B cell mediated immunity, positive regulation of B cell activation, B cell proliferation and B cell differentiation are displayed as heatmaps. Differential age- and genotype-associated gene expressions are shown as heatmaps or of selected genes, respectively in total spleen (**A, B**) and in blood (**C, D**) of WT and *ApoE*$^{-/-}$ mice. Results represent means±SEM. Analyses were performed using ANOVA with Benjamini-Hochberg correction. Absolute numbers of signal intensities and statistics are reported in supplementary Table S1.

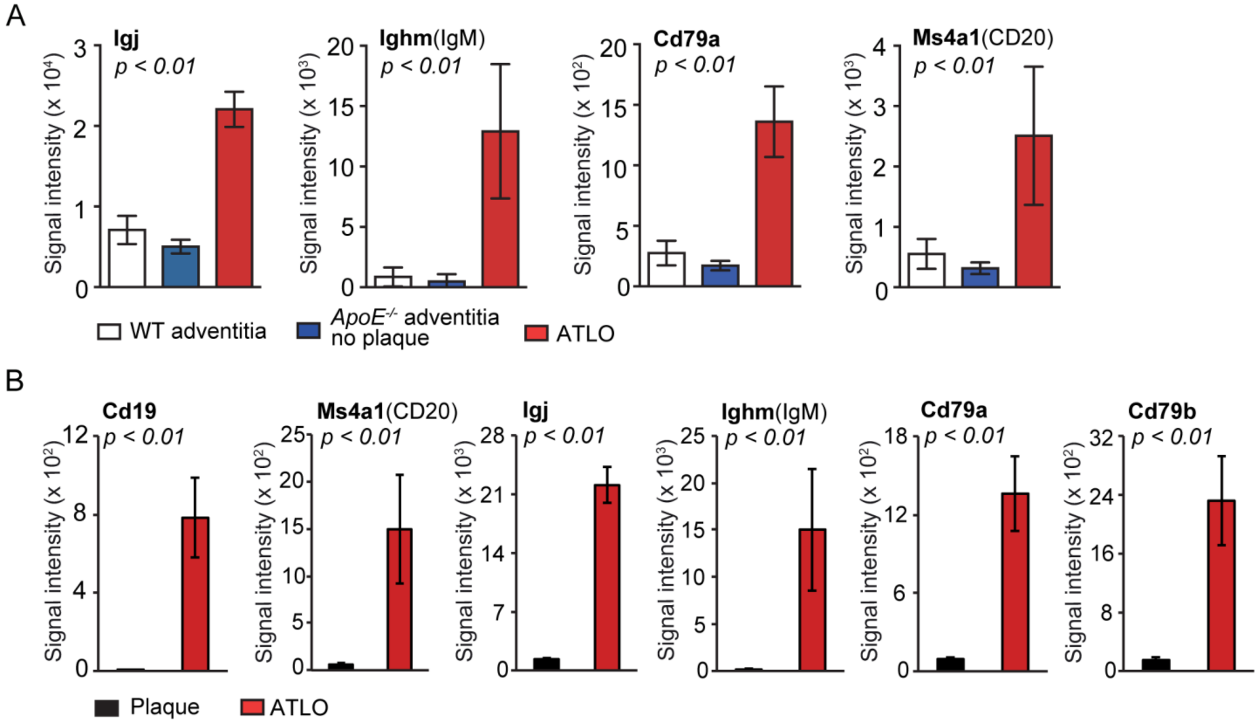

Figure SII: LCM microarray-based gene expression analysis of plaque versus ATLO. A*)* Differential expression of selected genes in adventitia cluster (WT n=3; $ApoE^{-/-}$ n=4) and ***B)*** plaque/ATLO cluster (for plaque $ApoE^{-/-}$ n=3; for ATLOs $ApoE^{-/-}$ n=4). Results represent means ± SEM. Analyses were performed using ANOVA with Benjamini-Hochberg correction. Absolute numbers of signal intensities and statistics are reported in online supplement Table S1 and online methods.

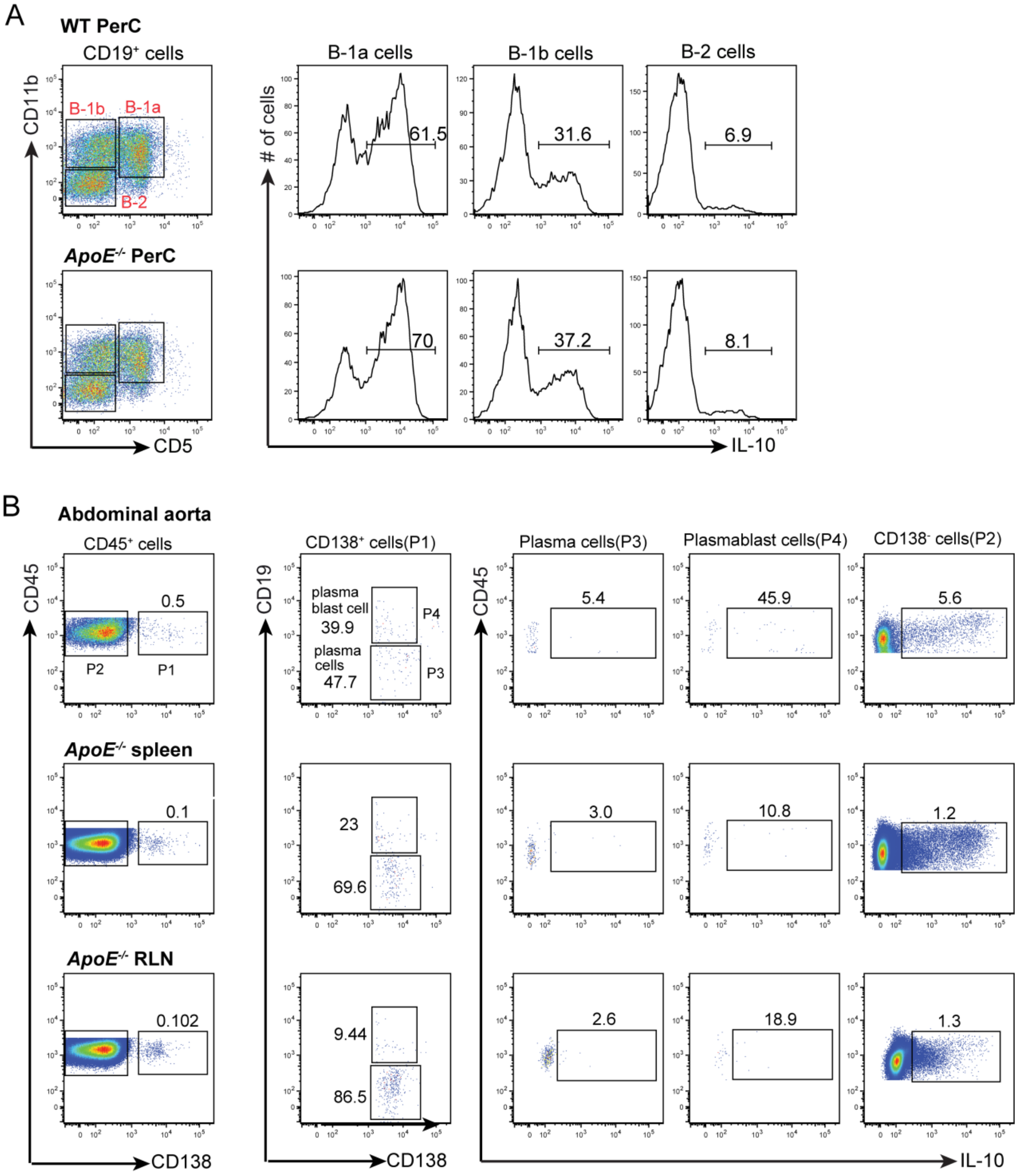

Figure SIII: IL-10 producing cells in perC, aorta, spleen, and RLN. Isolated cells from peritoneal cavity **A)** aorta, spleen and RLN **B)** of 80-85w old WT and *ApoE*$^{-/-}$ mice (n=3 mice per genotype) were stimulated as decribed in methods. Cells were stained with different antibodies as indicated. **A)** B-1a, B-1b, B-2 cells; **B)** PCs, plasmablasts, and CD138^{-} cells were gated and examined for IL-10 expression. Numbers on FACS plots are frequencies of positive cells per gate.

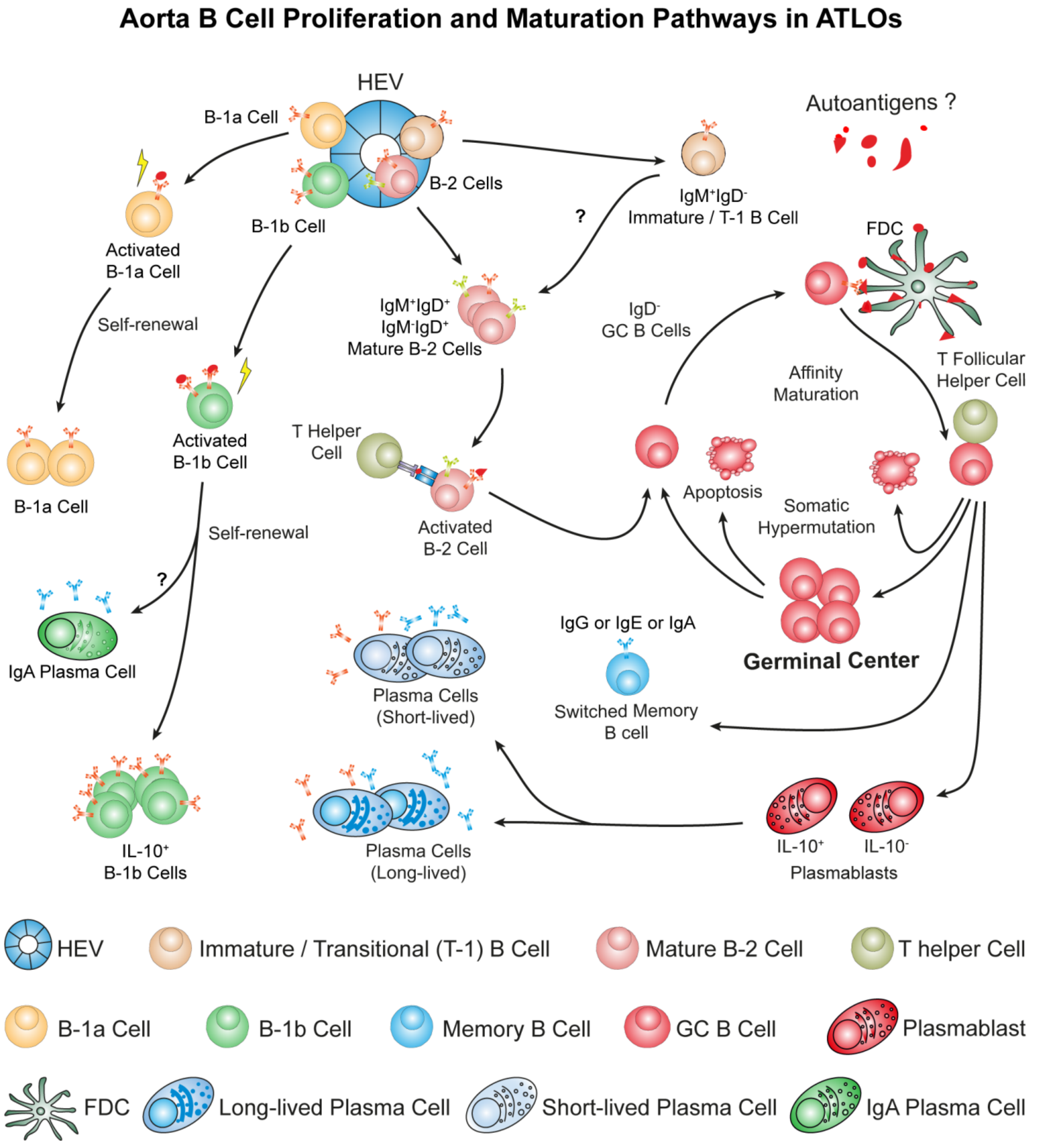

Figure SIV: Hypothetical choreography of ATLO B cell responses. Mature naïve B-2 or circulating B-1 cells enter ATLOs through high endothelial venules (HEVs). B-2 cells undergo T helper cell-dependent activation, proliferation, and maturation steps including somatic hypermutation and affinity maturation in germinal center reactions in response to arterial wall-derived autoantigen(s). The resulting high-affinity B-2 B cells either differentiate into memory B cells followed by isotype-switching and/or differentiation into plasma cells. A fraction of plasma cells expresses IL-10^+. B-1 cells are activated by inflammatory cytokines and - possibly in response to atherosclerosis autoantigens - undergo self-renewal to form IL-10^- B-1a or IL-10^+ B-1b cells. T cell immune responses except T helper cells and T follicular helper cells or antigen-presenting cells are not depicted for ease of reading.
